# Access Barriers to Services by Immigrant Mothers of Children with Autism in Canada

**DOI:** 10.1007/s11469-017-9732-4

**Published:** 2017-01-17

**Authors:** Nazilla Khanlou, Nasim Haque, Nida Mustafa, Luz Maria Vazquez, Anne Mantini, Jonathan Weiss

**Affiliations:** 1 Women’s Health Research Chair in Mental Health, Faculty of Health, School of Nursing, York University, HNES 3rd floor, 4700 Keele Street, Toronto, ON Canada M3J 1P3; 2 Critical Care Service Ontario, LuCliff Place, 700 Bay St, Suite 1400, Toronto, Canada; 3 Centre for Research on Inner City Health, Li Ka Shing Knowledge Institute, St. Michael’s Hospital, 30 Bond Street, Toronto, ON M5B 1 W8 Canada

**Keywords:** Autism, Canada, Disabilities, Immigrant, Mothers, Social support

## Abstract

Equal access for autism services remains suboptimal for diverse groups. In Canada, little is known about the barriers immigrant mothers face accessing services and support for their children with developmental disabilities. In this qualitative study, 21 immigrant mothers of children with Autism, from a diverse ethno cultural background, were interviewed in Toronto, Canada. We apply House’s (1981) four domains of social support to analyze findings. Structural support challenges, such as delays in diagnosis, fragmented and dispersed services were common, followed by instrumental challenges due to loss of social ties and stigma. Lack of expected support from partners, and negative perceptions of services, were identified as emotional and perceptive challenges. Focused attention is required to address inequalities within the context of current access pathways for autism.

Mothers of children with Autism Spectrum Disorder (ASD) require timely access to effective supports across service sectors, yet face many access barriers. Among immigrant families this may be further compounded due to acculturation stressors and learning to navigate new education, social and health sectors. Therefore, promoting equitable access requires that the challenges immigrant families face in accessing supports and services for their children with ASD be fully understood. Evidence for the role of social support in facilitating improved outcomes for both parents and children is consistent (Wang and Brown 2009; Hendricks, de Moor, Oud, and Franken 2000). In addition to accessing services, immigrant families of children with ASD require social support. Social support has been recognized as an important positive influence on the health and wellbeing of parents (WHO 2003; Pelchat 2007; Edmondson 2003), a way to reduce family burden for parents of children with disabilities (Lopez 2014), and has also been linked to the timeliness of and effectiveness in outcomes of ASD interventions (Osborne et al. 2008; Weiss et al. 2013). Both perceived and actual social support have been reported to reduce parental stress, increase coping skills, and prevent parents from developing conditions such as depression and anxiety (Samadi and McConkey 2014). If timely diagnosis and optimal intervention effectiveness partly depend on social support, the clinical access pathway to services for ASD needs to also consider the flow of support to parents through interaction with people and organizations.

Communities such as immigrant mothers with diverse levels of social support and access to services may also differ in their tendency to facilitate or delay diagnosis and intervention. Rates of ASD have shown to be 36% higher in children of immigrant mothers (Becerra et al. 2014). One study, for example, found that mothers born in Eastern Asia are 25% more likely to have a child diagnosed with ASD compared to non-immigrants (Dealberto 2011), and immigrants from Sub-Saharan Africa are 76% more likely (Bolton et al. 2014; Pavlish et al. 2010). Despite the evidence for the critical need to equal and timely access for children with ASD, very little research has been conducted to identify the challenges encountered by immigrant mothers (Fig. 1).

**Fig. 1:**
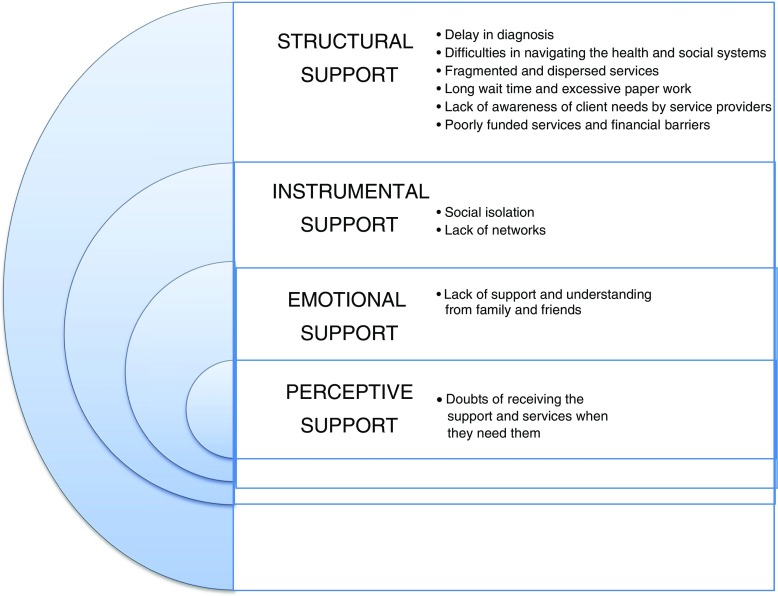
Social Support for immigrant mothers

In Canada, migration is an integral part of federal policy. In 2012 immigrants accounted for two-thirds of its population growth (Statistics Canada 2012). About 49% of Toronto’s population identify themselves as immigrants, one third being newcomers, having arrived in Canada between 2001 and 2011 (City of Toronto 2013). By 2018, over 60,000 refugee families from the Middle East, Nepal, Ecuador, Burundi, Ethiopia and Sudan will have re-settled in Canada. It is estimated that 56% of these refugees are families with children younger than 14 years of age – children who have not had access to either educational or health services for several years and parents with low literacy and language skills (Citizenship and Immigration Canada 2016). This increase in ethnic diversity of local communities impacts both the newcomers themselves and the host society with resultant implications for equitable social and health services access and provision.

Immigrant families typically leave behind strong social networks (i.e., close family and friends) in their home countries, but may bring with them perceptions and traditions from a society with different expectations for child development and available services. As a result, they may struggle to navigate through new systems of service for children, especially those with more complex developmental difficulties, such as ASD (Shepherd and Waddell 2015). There are also a host of variables that can impact the ability of immigrant families to access supports for their children and for themselves. Studies have documented that children of immigrants with ASD have reduced access to resources, early intervention and, thus, to positive outcomes (Chiao and Blizinsky 2013).

## Migration and Access to Services

Immigrant parents are often unable to access needed supports due to language barriers, low income, and transportation limitations (Luther, Canham, and Young Cureton 2005; Fellin et al. 2013). Additionally, inflexible service offerings by the host community, reduced social networks, lower employment and marginalization have also been reported to interfere with access (Greenwood et al. 2014). Further, the ways in which immigrant parents understand and approach childhood disability, parental acculturation levels, knowledge about child development and awareness of the availability of services, have been found to affect access to service among immigrant families (Alegria et al. 2011; Magana et al. 2013), contributing to their experience of social isolation, guilt and shame (Sorheim 2006). It follows then that parents in these situations may find it challenging to navigate across service sectors in raising their child who has been diagnosed with ASD.

Barriers to service access can begin even with regard to diagnosis for immigrant and non-immigrant families of diverse ethno cultural and racialized backgrounds. In the United States, the literature points towards racialized families receiving a later diagnosis for their child – the average mean age of diagnosis within these groups is reported to be 88–89 months (Daniels and Mandell 2013; Mandell, Listerud, Levy, and Pinto-Martin 2002; Shattuck et al. 2009). Canadian born children are most commonly diagnosed between the ages of 39 and 55 months, depending on the region in Canada where these children reside (Ouellette-Kuntz et al. 2009). The trend is particularly striking in the United States for autism diagnoses of mild/moderate levels of severity diagnosed who are more likely to be identified at significantly later ages for minority groups (Heejoo et al. 2015). This delay in diagnosis has been attributed to a number of clinical, socio-demographic and geographic factors (Daniels and Mandell 2013). There is also evidence for cultural factors, including poor understanding of rehabilitation potential (Miller-Gairy and Mofya 2015); family traditions, expectations of extended family and parents for child development based in traditions from their country of origin (Chiri and Warfield 2012).

The data on the prevalence of ASD in combination with increased influx and diversity of immigrant families will be reflected in a concomitant increase in the need for more timely ASD services. Therefore, understanding the nature of care needs and the experiences this population has in seeking the services their children is of paramount importance. Disparities in ability to obtain early diagnosis and intervention surface as a consequence of several factors but the role of social support is the least understood. However, it may also have the potential to reduce barriers to access. To improve access to social supports and services, social support itself and how it impacts on the clinical access pathway for children with ASD needs to be understood.

## Social Support

Social support is a multidimensional concept; a component of social interaction defined as an exchange of positive affect, a sense of social integration, emotional concern, and/or direct aid or services between two persons (O’Reilly 1995). House (1981) regards social support as an interpersonal transaction involving emotional concern, information, appraisal, or instrumental aid and refers to the aspects of support one receives from others. He offers four categorical dimensions of social support that permits categorization of social support used by respondents in relation to the type of support and allows for analysis of those which interfere or facilitate access to services. This approach further highlights the challenges for immigrant families and permits us to better identify what types of social support act as more effective facilitators of access to support and services for immigrant mothers of children with ASD. Firstly, applying House’s (1981) conceptualization of social support to our field of inquiry, *structural* support focuses on how social institutions (such as government immigration and social services, health care, and educational systems) in both policy and practice affect the quality of lives of immigrant mothers of children with disabilities. The next form of support is *instrumental*, defined as tangible support, such as provision of financial assistance, material goods or services. This form of social support encompasses the concrete, direct ways people in the community and friends and families assist others (Langford, Bowsher, Maloney, and Lillis 1997). *Emotional* and *perceptive* forms of support are more at the personal level, with emotional support focusing on partner/spouse’s support to help reduce the burden of care, and perceptive support referring to mothers’ perception of the adequacy and helpfulness of social support she feels are available to her.

Understanding the nature of developmental/social services/health/and education experiences and needs that immigrant families have in seeking services their children need is of paramount importance. To improve access to social supports and services, social support itself and how it impacts on the clinical access pathway for children with ASD needs to be understood. This paper presents findings from a larger study, *Mothers Project*, which explored the perspectives of mothers and service providers regarding social support needs, challenges and experiences of immigrant mothers of children with disabilities in Toronto, Canada. We present here the challenges that immigrant mothers’ of children with ASD encounter in accessing the needed social support and services. Access to services is critical for children with ASD and needs to be investigated by identifying the types of barriers mothers face in their efforts to obtain services and social support to promote their children’s healthy development.

## Methods

### Study Design

The *Mothers Project* applied a qualitative descriptive approach (Sandelowski 2010), conducting in-depth interviews to explore the experiences and perspectives of immigrant mothers regarding their access to social support (structural, instrumental, emotional, and perceptual support) (House 1981) in mothering children with disabilities. A qualitative approach was chosen because it is particularly helpful to generate information that is readily understood by participants and non-researchers, making the findings interpretable by general community, program managers and to help to increase the “volume” of marginalized voices and diversity of experiences (Sullivan-Bolyai, Bova, and Harper 2005; Ungar 2004).

The study was conducted in the Greater Toronto Area (GTA) between the months of April and December 2012. Purposive sampling strategy (Denzin and Lincoln 2011) was used to select participants through diverse service organizations in the GTA (general and snowball sampling). This type of sampling strategy allows researchers to select individuals and sites for the study that can provide specific information and understanding of the research problem under study (Creswell 2007). A recruitment flyer describing the study was developed and distributed to community partners and collaborators of the project. In addition an electronic version of the flyer was forwarded to the community partners and posted on websites. Ethics approval for the study was obtained from the University’s Research Ethics Board, Toronto, Ontario.

### Procedure

The interviewer who was also the project coordinator was trained by the principal investigator. After receiving calls from interested mothers, the interviewer called back each participant to explain the project, their role and the time required for the interview. At this point the interviewer would also try to schedule a time to conduct the telephone interview. To compensate for their time, participants were paid $30 after completion of the interview. All interviews were conducted by the same interviewer and at the beginning of each interview, the interviewer read the consent form, clarified any questions participants had, and obtained verbal consent from each participant.

### Inclusion Criteria

Potential participants were identified for the study using the following inclusion criteria:

(i) Immigrant mothers: only mothers who identified themselves as immigrants were interviewed
(ii) Potential participants who had one or more children with ASD. Mothers’ identification of her child/children having autism was accepted as confirmatory and no other documentation was used to confirm the diagnosis.
(iii) Potential participants who were comfortable speaking and understanding English language.

### Data Collection

Telephone interviews were conducted using a semi-structured interview guideline (Appendix 1: In-depth interview guide for mothers). The construction of the questionnaire was informed by existing literature (Harknett and Hartnett 2011; Miller, Gordon, Daniele, and Diller 1992; Skrinda 2008) and the principal investigator’s experience in working with immigrant populations. The questions were designed to explore the four domains of House’s classification of social support (House 1981) that addressed issues related to mothers challenges in accessing both formal and informal social support and services, school/education system, accessing informational and perceptual support, access to informal social networks such as professional or semi-professional support and if mothers had any advice for other immigrant mothers with similar background. Telephone interviews were completed in 60–90 min, and transcribed using Dragon Naturally Speaking software. Following each interview field notes were taken by the interviewer regarding her impressions about the interview. These notes were later discussed with the principal investigator and the research team during analyses meetings.

### Analyses

Analyses of the transcripts were completed by detailed independent review of transcripts by three members of the research team. Each researcher then independently generated themes and sub-themes which were later triangulated through face-to-face team meetings. The differences in the results by the three researchers were minor and related to choice of phrasing for themes, which were agreed upon through discussions in the meetings. A fourth team member then applied the selected themes and subthemes to remaining transcripts and corroborated with principal investigator regarding any new emerging themes and subthemes in relation to House’s (1981) four domains of social support.

### Rigor

Study rigor was enhanced through triangulation and member checking (Bennett, Deborah, and Allen 1996; Stainback and Stainback 1988). First, investigator triangulation was achieved by using multiple coders to analyze the findings; specifically analytical triangulation was carried out through research team meetings in which they discussed the emerging themes and clarified interpretations. Second, member checking was conducted by presenting our emerging findings at knowledge transfer events to mothers and service providers, and receiving their input on our findings.

## Results

Of the 30 immigrant mothers who participated in the larger study (Mothers Project), 21 mothers identified having one or more children with ASD. Table 1 provides summary information on the participants. Mothers had a total of 27 childrens with ASD and the ages of the children ranged between 2 and 31 years. Four mothers had two children with ASD and one mother had three children with ASD within this age range. Six of the children were between the ages of 6–9 years, six between the ages of 10–15, five between the ages of 15–20 and one child was an adult aged 31 years. The countries of origin of the immigrant mothers represented the continents of Europe, Asia and Latin American and the Caribbean. The ages of mothers ranged between 32 and 56 years.

**Table 1:** Characteristics of participants

Characteristics	Mothers *n* = 21 (70%)*
Age in Years	
Mean	41.3
Range	32–56
Marital Status	
Currently married	19
Divorced	2
Years lived in Canada	
Mean (years)	10.8
Range (years)	0.75–23
Region of origin	
Asia (China, Philippines, Taiwan, Japan, India, Pakistan, Sri Lanka)	14
Europe (Ukraine, Poland, Belgium, Romania, Albania)	5
Latin America (Costa Rica, Trinidad And Tobago) and Tobago)	2
Number of children per family	
Mean	2.2
Range	1–5
Children with ASD	
Total number	27
Age range (years)	2–31
Gender of Children with ASD	
Boys	16
Girls	11
Age of diagnosis	
Mean (years)	3.7
Range (years)	2–12

The findings of the study are presented below and according to House’s four dimensions of social support (i) structural, (ii) instrumental; (iii) emotional and (iv) perceptive (House 1981).

### Structural Support

Mothers raised a wide range of concerns related to structural support. As presented below these included (i) delay in getting definitive diagnosis of children, (ii) navigating the system, (iii) fragmented and dispersed services, (iv) long wait time and excessive paper work, (v) lack of awareness of client needs by service providers and, (vi) financial barriers and transportation challenges as subcategories of structural barriers that hindered mothers from accessing services for their children. The following are examples supported with mothers’ quotes; personal identifiers of mothers are removed to maintain anonymity.

#### Delay in Diagnosis

Mothers in our study discussed the issue of delayed definitive diagnosis of their children and its implications in accessing government and other resources. One mother (Mother 25: M25) explained that her child was 1.5 years old when he was first identified with having a global developmental delay. She further stated it was just a *“so and so diagnosis”* meaning they were *“not very sure”.* She explained the second diagnosis they received at the age of 5 years as autism which was a definitive diagnosis but by then *“I was sure what the problem was already”* (M25).

Another mother explained the implications of delayed diagnosis in these words:

One thing - autism is different. No diagnosis and you can’t access services or funding from the government. No disability tax credit. (M17)

Almost all immigrant mothers wanted to have a confirmed diagnosis of their child at an earlier age. Uncertainties about their children’s conditions aggravated their stress. Therefore, receiving an early diagnosis may help mothers to focus on understanding and learning about the disability of their child as well as on taking decisions as to how to face the situation.

#### Navigating the System

Several mothers in our study discussed the challenges they faced because of lack of information about the resources available for their children. This lack of knowledge delayed their access to services. One mother expressed her concern in these words:

We have to do all search about their [ASD children] services by ourselves. Lots of work, especially for immigrants. (M9)

Communication barriers and not understanding medical terminologies may further aggravate parents’ frustrations. Many mothers in our study did not understand the meaning of “respite care” as pointed out by service providers, and therefore, were not able to request for these services even when they were available. The lack of such support in the system creates barriers to these families trying to seek help. Findings illustrate that even when services exists, immigrant mothers may not actually have access to them; as it is discussed further, there are different factors mediating and preventing immigrant families from access to services.

#### Fragmented and Dispersed Services

Almost all mothers reported challenges they face in accessing services which are scattered all over the city. Participants reiterated that it was very difficult for immigrant mothers, especially recent immigrants, to locate the service locations and to find transportation to take their children in a timely manner. They further emphasized their dissatisfaction on the discordance between the health, social services and the school system:

The system is very fragmented. The school does not speak about social services. No combined packages. (M17)

Services are scattered everywhere. It took me 3 or 4 months to figure out what to do. I don’t know ….so many services …… I don’t know if I could apply for them or what to do with them. A lot of waste and redundancy in term of resources. Better management. Integration of resources. If you are lucky you run into a good lead. I don’t know if they designed it this way to serve only a few people at a time. (M28)

The best was …[name of centre]. It’s a wonderful place. They offered speech, occupational therapy, psychologist services. There was a long list at first but you are discharged by the time your child is six [year old]. (M5)

Lack of integration and coordination of the services provided by governmental and non-governmental organizations from different levels of government (local, provincial, national) make the system complex and difficult to understand for newcomers and immigrants unfamiliar with Canadian institutions.

School is an important aspect of children’s life. Almost all mothers in our study spoke about their frustration and the challenges they encountered with schooling of their children:

The schools are reluctant to give kids support - a lot of kids have problems and are put into the lowest class with a developmentally delayed program which is like babysitting. It’s like daycare. (M2)

Where can I put my child when he’s not being accepted in school? (M24)

#### Long Wait Time and Excessive Paper Work

Access to programs is time intensive, as there may be long waiting lists before families are able to utilize the services. Most mothers expressed their frustration regarding long wait times for accessing services and more importantly not knowing when their child’s turn will come:

Wait list in north of Toronto is 4 years. I got on in 1 year and 3 months in my area. Who organizes the wait list and how? Everything is hidden. There is no visibility. No access to the end of the day. Not helpful. (M26)

In the GTA there was a long wait - 18 months for the developmental pediatrician. That is just unacceptable at the age of 4 or 5. (M17)

Mothers reported that when they receive the service it is sometimes too late or the potential benefits of the services, such as speech therapy, are diminished when the children receive the service at an older age. Other times they may receive the phone call from agencies when they are away from their homes, missing the opportunity to accept the service so they need to register again in waiting lists. Excessive paper work was also identified as a major barrier in accessing services. Findings show that mothers are discouraged by complicated bureaucratic process to get services, such as the completion of long forms that are difficult to understand.

#### Lack of Awareness of Client Needs by Service Providers

Many participants in the study expressed their concerns about inadequate awareness regarding the needs of children with disabilities even among some professionals:

One doctor was useless. More education amongst service professionals is needed. I got help from others, not professionals. Then you go to professional to tell them what it is I need. It’s the wrong way around; maybe this is a systemic problem. (M8)

I have to say some pediatricians are not supportive. They have been practicing a long time – they need to be updated. They are not a good resource. She asks questions, checks the height. We need someone who’s helpful and gives information. (M10)

In general mothers were satisfied with the services of the family doctor. However, some mothers expected their family doctor’s office to be more sympathetic to their situation and provide emotional support to help them cope with their crisis. They also expected their family doctors to provide more information on service availability for their children.

#### Poorly Funded Services and Financial Barriers

Mothers underlined their inability to bear the financial costs of many of the tests and services that are not covered by government but are required for the proper diagnosis and/or development of their children. Some blamed the government for not providing sufficient funds into the programs leading to long wait times. In many instances parents had to buy the expensive services for their children. The frustrations of mothers are evidenced by the following quotes:

[They say] it’s not my problem - there are no funds. Can I tell my son there are no funds? I felt disempowered. I had no job. How do you stay positive? (M26)

Services are being paid for….in order to get into programs you have to be assessed and that costs money and their programming is for the elite, it costs money to get into their programs. People stopped going. (M2)

Lots of people are unable to afford counseling as it’s very expensive. (M5)

The struggles immigrant mothers endure to have access to services are embedded in a context of economic hardship. Usually mothers give up their job to be able to take care of their children with ASD, and in many cases new immigrant fathers have unstable low pay jobs.

### Instrumental Support

Instrumental support is tangible support that mothers can get from formal networks such as family and friends, and from informal networks, such as social institutions and compassionate service providers. However, many recent immigrant mothers do not have these supporting networks in their new country of resettlement. Some immigrant mothers can face stigma due to their child’s disability from their own extended family and friends that they depend on. Thus, taking care of a child with autism and at the same time trying to adapt to a new culture without helpful social support, increases the burden of care for these mothers. This process can lead to social isolation of mothers.

#### Social Isolation

Many mothers reported feeling socially isolated because of the disability their child has, which is consistent with previous findings (Jennings et al. 2014; Woodgate et al. 2008). For example mothers expressed these challenges in the following words:

In 10 years in Canada I have never heard of a support group for mothers with children with disabilities for immigrants. (M3)

There should be a place where immigrant moms should be able to go to get something to do - like the job search workshops - but for moms with kids with disabilities. (M24)

Sometimes it’s hard to speak with people who do not know about autism. It’s hard to talk to family and friends. I kept it a secret….. (M23)

I’m home alone all day. It is very isolating – very. (M17)

Social isolation is also a main driver of feelings such as low-esteem and frustration among immigrant parents. Feelings of being judged by their extended family, about the way they raise their children with developmental disabilities, further contributes to mothers’ isolation.

### Emotional Support

Emotional support may be offered by close family members in particular by partners. However, many immigrant mothers face emotional challenges in terms of lacking support and understanding from their own family units.

My husband worked all the time. He is pretty much not available. He went into withdrawal… At this time, I am on my own completely - I had bills, no support - waited till the government gave $100 per month… (M8)

[Husband] he is not involved at the same level, he leaves it to me. (M2)

The loss of social networks and social support from extended family and friends upon immigration to Canada was also an important concern expressed by mothers:

As an immigrant I had no family, I had a few friends but we moved a lot. However, I made a couple of friends in a parent group and I am still friends with them…. One of the most important things is to speak to others in the same situation. (M6)

### Perceptive Support

Perception of support refers to an individual’s view on whether the support provided is sufficient and helpful. Some mothers in our study expressed their doubts of receiving the support and services when they would need it.

I have no government services yet! I was not guided properly…there is no system to guide you. (M7)

We need more respectful translators with the knowledge about disability. (M16)

The system is unfair… they should check how much each family needs. So bigger number of families can access funding. (M10)

Findings illustrate that the existence of services in itself does not necessarily translate into solving immigrant mothers’ problems. Mothers’ perceptions of usefulness of services, their appropriateness, and timeliness are also key to understand factors mediating service use. As one mother stated, *“justice delayed is justice denied”* (M3).

## Discussion

The literature indicates the need for an increase in awareness of multicultural and immigrant experiences regarding ASD (Dyches, Wilder, Sudweeks, Obiakor, and Algozzine 2004; Singh et al. 2013), however, there are few ethno cultural specific studies in Canada that highlight the specific challenges immigrant mothers encounter in accessing support and services for their children with ASD. This study is an important step toward this direction. Our findings illustrate two processes that may influence immigrant mothers’ access to social support and services. The first is the process of migration and settlement that embeds socioeconomic constraints, language barriers, lack of social networks, and acculturation challenges. The second is related to the quality and existence of social services for immigrant mothers that according to the mothers’ narratives are limited, non-existent or difficult to access. These two processes are closely intertwined, as they appear interconnected in the everyday lives of immigrant mothers. For example, experiencing difficulties in access to services may accentuate immigrant mothers’ lack of social networks, and vice versa. Lacking access to public services and having to pay for private services may contribute to the socioeconomic burden of immigrant families.

### Structural Support: Migration and Settlement Challenges

Immigrant mothers face structural barriers related to migration and settlement processes. In Canada recent immigrants live below the poverty line, have three times more probability of living in poverty than people born within the country, experience unemployment, occupy low pay and precarious jobs, experiencing “earning and occupational disadvantage[s]” (Reza and Kazemipur 2013; Affiliation of Multicultural Societies and Services Agencies of BC 2013). The expenses these families encounter in regards to finding and accessing the necessary care for their child are challenging (Wang and Casillas 2013). Some programs tailored for children with ASD may require additional funds that can be expensive for parents, especially those who may be facing other migration expenses (Ennis-Cole et al. 2013) and do not work full-time employment with benefits. For newcomers with precarious jobs (unstable, low pay) there may be material implications in the way families care for their children. It is in this context what may appear as simple tasks such as attending medical visits and doctor appointments, may become a challenge for these mothers due to transportation limitations (Welterlin and LaRue 2007).

Findings from this study illustrate disparities in navigating the health and social systems in Canada. Language and complicated service delivery systems, communication, and social differences may create barriers to parents trying to navigate through new and unfamiliar service systems. Many new immigrant mothers in the GTA have English as their second language and may not have the strong social networks and family ties that they used to have in their home countries. This limits their ability to readily gain information and seek help. Previous studies have suggested the importance of educational programs for parents as a way to give information in a more interactive way, reducing parental stress and increasing their quality of life (Chiang 2014; Hoogsteen and Woodgate 2013; Kvarme et al. 2016; Tung et al. 2014). Knowledge to navigate across systems requires a set of structural conditions (language, access to networks), and availability of appropriate services for immigrant mothers as well (Khanlou et al. 2015; Asanin and Wilson 2008; Stewart et al. 2006).

Social isolation may be associated with both the structural socioeconomic conditions of immigrant families as well as with the societal stigma mothers encounter when caregiving their children with ASD. Similar to Canadian-born mothers of children with developmental disabilities, we found that immigrant mothers are the ones who often quit their jobs to take full responsibility for their children’s care, while fathers continue to work. The lack of paid employment represents not only an economic burden but also a factor some mothers perceive that exacerbates their isolation from their former networks and social life. Mothers feel isolated and frustrated due to the lack of employment, or limited employment conditions that allow them to work part time and have flexible schedules to care and advocate for their children.

### Quality and Access to Services

Families of children with ASD rely on more than one method or service sector in order to plan a successful education and integration plan for their child. Parents need to partake in a broad range of services simultaneously, in order to support their child in different ways (such as Speech and Language, Occupational therapy, and Behavior Therapy). However, newcomer parents use such services in different, more limited ways, than non-immigrant families. They also tend to access these services at a later age during their child’s life. Research in the United States indicates that ethnic minorities are half as likely to use a case manager for their child with autism, and only a quarter of this group visited a psychologist or developmental pediatrician in the early years (Thomas, Ellis, McLaurin, Daniels, and Morrissey 2007). Access to care was highlighted as one of the limiting factors for minority groups unable to receive adequate services.

Social support in terms of knowledge, access, and utilization of services is often limited within immigrant communities. Challenges to navigate the social and health systems may be a signal of the need to provide more individualized and family centered programs and services; a clinical access pathway that also accommodates the needs of immigrant mothers. Stewart et al. argue that “[s]ervices may not be linguistically or culturally appropriate because they are often predicated on Euro-Canadian standards of care” (2006, p. 336). The challenges the service delivery system faces in terms of providing culturally sensitive services are complex, especially in the Canadian context where the population is increasingly ethno-culturally diverse (Khanlou et al. 2015). Further, there is the complexity of tradition where some cultures will not acknowledge that children have developmental disabilities because it is not culturally acceptable. The lack of familial and community support contributes a great deal to how mothers are able to deal with the diagnosis. For many the ASD diagnosis is difficult for fear of their child experiencing the negative consequences that would have been typical in their country of origin (e.g., stigmatization, removal, seclusion).

An especially important finding from this study is that immigrant mothers are rarely asked about their needs and about what services are best for them. This is particularly important in the immigrant family context because of the diverse values and belief systems mothers from different backgrounds. Mothers’ traditional beliefs and values may influence how they follow up with treatment and professional supports (Kummerer, Lopez-Reyna, and Hughes 2007; Smith 2011). Further research is needed to identify immigrant families’ views regarding the type of services that best fit their needs. One potentially fruitful endeavor would be to examine how child welfare agencies may assist in creating and implementing family-centered service plans. Immigration is increasingly recognized as being an important factor in child welfare (Maiter, Stalker, and Alaggia 2009), and can be a critical resource for immigrant families and can be an important source of social support for immigrant mothers (Urquia et al. 2007). Social supports available through child welfare agencies may be emotional, instrumental or informational and can cross a broad spectrum. Maiter et al. (2009) suggest that child welfare agencies advocate for the provision of more instrumental social supports, such as more accessible English as second language (ESL) classes and greater levels of support for immigrants seeking entry to the work force. In the case of immigrant mothers who live with extreme hardship and heavy stressors (Urquia et al. 2007), child welfare or even settlement services may take on a heightened importance for mother and child well-being and safety.

Mothers in our study spoke of social isolation and little leisure time but also infrequent contact with friends and family. Consistent with previous research (Greenwood et al. 2014), the new immigrant mothers in our study had limited knowledge about how to obtain help, who could help them and what their rights were in caring for a child with autism. For immigrant families, efforts should be channeled to promote policies that extend welfare and settlement services to health care clinics and doctors’ offices, community based organizations, and child care centers.

## Limitations

The findings of this qualitative study should be considered with certain limitations in mind. *First*, participants self-selected to participate in the study from a limited geographical area and may not necessarily be representative of all immigrant mothers of children with ASD in Canada. Different findings may emerge in other contexts depending on participants’ cultural, racial and ethnic background, income level, language skills, and migration status. Future research should focus on comparative research between ethno-cultural groups on the effects of acculturation stressors and resettlement supports on mothers’ experiences with school integration, developmental and health services, as well as differences in their adaptation to raising a child with ASD. However, the goal of this qualitative research was not to statistically generalize the findings, but to explore in-depth the challenges immigrant mothers of children with ASD encounter in accessing services, and to generate knowledge and understanding about their lived experiences that can be made transferable, as per a qualitative approach (Khanlou et al. 2008). *Second*, another limitation stems from our inclusion criteria to select mothers with some comprehension of English language, therefore the experiences of mothers with significant language barriers may have been different and were not captured in this study. *Third*, another comparative limitation is the lack of inclusion of fathers in our study. Research suggests that husbands are the main source of support to the mother. Having a husband is also a significant predictor of stress and adjustment in mothers (Bennett et al. 1996; Bristol, Gallagher, and Schopler 1988). Other studies have shown that extended family members and friends also play an important role in providing advice and information to mothers. However, family members are described as more important sources of material and physical support, including respite care (Bennett et al. 1996; Welsh et al. 2014). Paradoxically, family members could also be a source of stress for mothers if they were perceived by mothers to be judgmental and critical of their caretaking skills.

## Conclusion

As prevalence of autism has risen sharply, so has its visibility. Consequently, the role of parental and community resources for early identification and intervention for children with ASD has improved overall. However, for some groups, access continues to be limited by both individual and community-level factors that may affect the age of diagnosis and follow-up therapy. Unique challenges are experienced by immigrant mothers in accessing the social support and services they need for their children, such as language barriers and navigating new service systems. Compounding their stressors is when these families face additional socioeconomic challenges due to underemployment or precarious employment of parents. In sum, child developmental disabilities can be emotionally and mentally exhausting for all communities, but are further complicated for newcomers.

The intersection of immigrant status (legal, precarious) and access to services (health, housing, school) in Canada is critical in understanding whether and how immigrant families are accessing them (Goldring and Landolt 2013; Villegas 2013; Asanin and Wilson 2008). The existence of services for immigrant families does not necessarily mean or guarantee that they access and use them. Some immigrant mothers believe that if they ask for help, they will lose their immigrant status, so they do not seek help or support.

The challenges faced by immigrant mothers of children with autism need to be urgently addressed in order to facilitate effective access to needed social support and services. Further, to inform policy and design cost effective interventions at the community level, prevention efforts must be informed by an understanding of mothers’ social support and service needs. Targeted policies and practices (such as increasing availability of multi-language materials, adequate interpreter services, raising culture-specific awareness of the importance of early diagnosis and intervention, and raising knowledge of Canadian disability accommodation rights/laws) may have an impact on the pathway to diagnosis and treatment of ASD, better accommodate the needs of immigrant families, and support health equity.

## Appendix 1: In-depth interview guide for mothers

Demographic Questions:

1. Can you please provide your date of birth?
2. Are you married, widowed, single, separated or divorced?
3. Where were you born?
4. How long have you lived in Canada?
5. How many children do you have?
6. What are the sexes and ages of your children?
7. Can you tell me a bit about your children?
8. How long has it been since your child was first identified as having a disability?

Interview Guide

Structural Support

I am first going to ask you a few questions about health care

9. Does your child have a doctor? Is it a paediatrician or a family doctor or both? Can you understand your child’s doctor?
10. Do you agree with the diagnosis your child has and if not, can you please explain how your child(rens) condition has affected you and your family?Aa
11. Do you attend clinics at a hospital with your child? Do you find there are supports offered through these clinics?
12. What has been your experience of hospitals/doctors in Toronto (GTA)?
13. Are you physically able to manage your child’s needs? If not how do you cope?

I am now going to ask you a few questions about your child(rens) education

14. Does your child attend school?
15. Have you found the school to be supportive of you and your child? Has there been any disagreement with respect to your child and his or her schooling? Have you been through an IRPC process with your child who has a disability? If so, did you attend an IPRC on your own?
16. How would you describe your experience with the special education department at your child’s school?
17. Did you feel at the time of identification for special education services at the school that you had the support of your family and friends?
18. Do you feel understood by your child’s teachers? If not, can you please share with us why not?

Informational and Perceptual Support

I am going to ask you questions about other supports and services for your child(ren) with disabilities

19. Do you face any difficulties in obtaining items that your child needs? If yes, how does this impact on you?
20. Do you use any government services to assist you with the care of your child? If so which ones? Do you find these programs beneficial to you? In what way are they supportive and how could they be more so?
21. What is the name of the service and can you tell me about your experience with the service you use? (prompts – enough time allotted to your family? Language issues? scheduling issues? Other experiences you can provide information to me about).
22. Are there any resources or supports that you do not have that you feel would be supportive to your own well-being that we have not talked about here today? For example, in the country you come from originally were there supports there that are not available here in Ontario?
23. Can you afford to meet all of the needs of your child(ren) with a disability? For example, have there been interventions or tests that you have wanted to get for your child(ren) but been unable to afford them (psych-ed evaluations, drugs that are not covered by drug benefit plans).
24. How have you dealt with that?
25. Do you receive any funding from any of the Ontario government programs for children with disabilities?
26. If not, have you tried to access them? What happened? What was the impact on you?
27. Do you understand what benefits you are entitled to/not entitled to? For example, are you aware of the child disability child tax credit? Do you have someone or someplace you can go to find out information about such things?

Emotional Support

I will now ask you a few questions about emotional support

Informal social network

28. If you are married, or in a long term relationship does your husband or partner support you in the care of your child/children? In what ways does he or she support you?
29. Is the level of support he offers “enough”? Does he have the same understanding of your child’s needs as you do?
30. Do you have other family members who help you with your mothering? In what ways do they help you?
31. Is there ever any conflict with respect to the help you receive from family members? Is family member support “ideal”? Who would the best person be in your life to be in this role?
32. Does your child with a disability have a good relationship with the family member who most supports you as a mother?
33. Do your family members accept your child?
34. Do you have any friends who have a child with a disability?
35. If yes, do you find your friend is supportive to you with your situation? Is it a mutually supportive relationship?
36. In what ways are they supportive to you and you to them?

Professional or semi-professional support

37. Do you belong to a support group for mothers?
38. If yes, how did you find out about this group?Aaa
39. Do you participate at any disability related groups or organizations which offer services, supports or programs to you and your child with a disability? (Like March of Dimes or Easter Seals, Geneva Centre for Autism, Ontario Association for Cerebral Palsy etc.).
40. Do you belong to any community organizations that you would describe as being supportive? Which ones?
41. Can you share a bit about your experiences with the level of support and the nature of the support you are provided by these organizations?
42. If you belong to a religious community does it provide social support to you? How big a role and in what way? What ways does this impact you?
43. With respect to the support you receive from your friends (if any) can you count on them all the time, some of the time or just occasionally? How does this impact you?
44. With respect to other social support, can you count on it all the time, some of the time or just occasionally? How does this impact you?
45. Do you have time to rest and or relax? How much time do you estimate that you have to yourself in a given week?
46. Do you have social time with friends and family? If not, why not?
47. Do you feel supported by the health and social service sector? How would you describe your experience with the health and social service sector?

I am now going to ask you questions about strategies and advice you may wish to share with us

48. Do you have any strategies that you have developed to make your life as an immigrant mother (with a child with a disability) run more smoothly and that you would like to share with other mothers?
49. Do you have any advice for other immigrant mothers with children with disabilities? (for example how best to advocate for your child effectively given your immigrant status and the barriers you have faced and described).
50. Is there anything else that you would like to tell me?
